# BCG vaccination and tuberculosis prevention: A forty years cohort study, Monastir, Tunisia

**DOI:** 10.1371/journal.pone.0219991

**Published:** 2019-08-05

**Authors:** Cyrine Bennasrallah, Meriem Kacem, Wafa Dhouib, Imen Zemni, Manel Ben Fredj, Hela Abroug, Amira Djobbi, Assia Green, Samia Grira Said, Issam Maalel, Sarra Stambouli, Wafa Zhir, Hichem Bel Haj Youssef, Asma Sriha Belguith

**Affiliations:** 1 Department of Epidemiology and Preventive Medicine, University of Monastir, Monastir, Tunisia; 2 The Regional Direction of Primary Health of Monastir, Monastir, Tunisia; 3 Department of Family Medicine, University of Monastir, Monastir, Tunisia; Fundació Institut d’Investigació en Ciències de la Salut Germans Trias i Pujol, Universitat Autònoma de Barcelona, SPAIN

## Abstract

We aimed to describe incidence, trends of tuberculosis (TB) over 18 years and to evaluate the impact of the BCG vaccine after four decades of immunization program according to three protocols. We performed a cohort study including declared cases in Monastir from January 1, 2000 to December 31, 2017. We reported 997 cases of TB. The predominant site was pulmonarylocalization (n = 486). The age standardized incidence of pulmonary and lymph node TB per 100,000 inh were 5.71 and 2.57 respectively. Trends were negative for pulmonary TB (PTB) (b = - 0.82; r = -0.67; p<10^−3^) and positive for lymph node localization (b = 1.31; r = 0.63; p<10^−3^). We had not notified cases of HIV associated with TB. Crude incidence rate (CIR) of PTB per 100,000 inh was 8.17 in Non-Vaccinated Cohort (NVC) and 2.85 in Vaccinated Cohort (VC) (p < 0.0001). Relative risk reduction (RRR) of BCG vaccination was 65.1% (95%CI:57.5;71.4) for pulmonary localization and 65% (95%CI:55; 73) for other localizations. We have not established a significant RRR of BCG vaccination on lymph node TB. Protocol 3 (at birth) had the highest effectiveness with a RRR of 96.7% (95%CI: 86.6%; 99.2%) and 86% (95%CI:71%;91%) in patients with PTB and other localizations TB respectively. In Cox regression model the HR was 0.061 (95% CI 0.015–0.247) for PTB and 0.395 (95% CI 0.185–0.844) for other localizations TB in patients receiving protocol 3 compared to NVC. For lymph-node TB, HR was 1.390 (95% CI 1.043–1.851) for protocol 1 and 1.849 (95% CI 1.232–2.774) for protocol 2 compared to NVC. Depending on the three protocols, the BCG vaccine had a positive impact on PTB and other TB localizations that must be kept and improved. However, protocols 1 and 2 had a reverse effect on lymph node TB.

## Introduction

Background: Tuberculosis (TB) is one of top 10 causes of mortality in the world, responsible for 1.7 million deaths in 2016 [1]. Over 90% of TB cases occur in low and middle-income countries [1].

The TB epidemic was perceptibly controlled in the late 1960s favored by effective antibiotic treatments and global mobilization against the disease. In recent years, the number of cases has been rising, announcing a threatening return of TB. In fact, experts from the WHO estimate that one-third of the world’s population is infected with TB, that every year 8 to 10 million new cases develop and that 3 million deaths are due to TB [2].

This recrudescence is accompanied not only by an increase in the number of antibiotic-resistant strains, but also by a human immunodeficiency virus (HIV)-TB coinfection [3]. The HIV pandemic presents a massive challenge to the control of TB at all levels. In high HIV incidence population, TB is a leading cause of morbidity and mortality, and HIV is driving the TB epidemic in many countries, especially those in sub-Saharan Africa [4]. TB trends have not been affected by HIV epidemic in Tunisia. Indeed, Tunisia is a low HIV incidence country [5].

In Tunisia, TB control efforts began in 1956 as mass screening campaigns. In 1979 the National TB Control Program [6,7]was launch including:Vaccination against TB: Bacillus Calmette-Guerin (BCG) and systematic screening for TB among patients with suspicious symptomatology.

Consequently, the incidence of TB decreased from 48.8 per 100,000 population in 1976 to 22.5 in 2007[6]. But then, an increase has been noted again with a 38/100,000 inhabitants inh in 2017 especially at the expense of lymph node localizations [8]. From 2010, the TB surveillance system in our country has been based on the registration of cases for treatment. BCG vaccine played a key role in reducing mortality rate and serious neurological complications [9].

BCG is believed to protect children from acquiring Mycobacterium tuberculosis infection[10] and from developing severe forms of TB disease such as TB meningitis and military TB [11]. It has been forty years since the launch of BCG vaccine in Tunisia. Despite extensive research, the exact BCG induced protection and efficacy are still debated.

The population-level effect of BCG vaccine varies between countries, depending on the vaccine used, implementation strategies, and vaccination coverage achieved.

Objective: Our study aimed to describe incidence and trends of TB, over a period of 18 years and to evaluate the impact of four decades of vaccination.

## Methods

### Study design

We performed a cohort study during 18 years (2000–2017).

### Setting

Monastir is situated in the coastal region of Tunisia. It is a governorate with university, touristic and industrial vocation and with a wealth of olive oil. In 2014, the general population of Monastir governorate was 548 828 inhabitants, representing 4.99% of the Tunisian population [12].It includes 13 delegations (Békalta; Bembla; Beni Hassen; Jammel; KsarHellal;KsibetMédiouni; Moknine; Monastir city; Ouardanine; Sahline; Sayada; Teboulba; Lamta-Bouhjar).

The national immunization program has been implemented in all governorates of Tunisia including Monastir. The year 1979 marked its start. Vaccination against TB included three periods with different protocols. In protocol 1, adopted from1979to 1995,the first dose was administered at birth followed by other injections at 6, 12 and 18 years of age. In protocol 2, adopted from 1996 to 2005,vaccinations at 12 and 18 years old were removed from the program. Protocol 3; adopted from 2006 to 2017, the 6-year injection was also removed;a single dose is administered at birth.BCG vaccination coverage achieved 91% in Tunisia [13]. According to national guideline TB management, declaration of TB disease in Tunisia is mandatory since 1959. Therefore, all public or private health physicians, general practitioners or specialists, first or second line hospitals should report all diagnosed cases of TB.

A TB case was defined as a notified bacteria-positive (smear-positive or culture-positive) and/or clinical and histological findings compatible with TB. All patients with confirmed TB were recorded in a database in the Regional Direction of Primary Health of Monastir (RDPH).The RDPH is the responsible of regional surveillance system. Itcollects the data of mandatory declarations of all delegations in Monastir Governorate.

Department of Preventive Medicine and Epidemiology in University Hospital of Monastircollaborates with the RDPH for data analyze and consequent prevent actions. The RDPH is the only regional source of TB drug supply. Distribution of drugs was carried out only after confirmation and declaration of cases.

### Participants

We analyzed all TB cases, residents in Monastir Governorate, declared to the regional direction of primary health care from January 1, 2000 to December 31, 2017. Cases living in another governorate were not included.

### Variables

We includeddemographic variables (age, sexes), vaccination status, year of diagnosis and TB localizations. Before 2006, the electronic database encoded TB localizations as pulmonary or extra-pulmonary, then since 2006 variable was encoded according to each specific localization.

### Data sources/Measurement

Data were collected in the RDPH of Monastir.For vaccination status, we considered Not Vaccinated Cohort (NVC) the population born before 1979, those born after as Vaccinated Cohort (VC).Thus, three cohorts were defined: VC (aged 0–23 years), transient cohort (aged 23–37 years) and NVC (aged more than 38 years). TB is a potentially serious infectious disease caused mostly by *Mycobacterium tuberculosis*,(*M*. *tuberculosis*) and *Mycobacteriumbovis (M*.*bovis)*. We used the WHO classification systems to categorize the sites of TB disease into pulmonary and extrapulmonary[14].

Diagnostic criteria were, according to WHO, for pulmonary tuberculosis (PTB): Two or three initial sputum smear examinations positive for AFB (acid-fast-bacillus), or one sputum smear positive for AFB plus radiographic abnormalities consistent with active PTB, as determined by a clinicianor, cases with three sputum smears negative for AFB but clinical and radiological features compatible with active TB.Lymph-node TB was considered as the infection of lymph node by TB. We note that other TB localizations include pleural, digestive, genitourinary tract, skin, articular-bone, meninges, ocular, renal, and central nervous meningitis TB (localizations other than lungs and lymph nodes).We defined extra-pulmonary tuberculosis (EPTB) as some of lymph-node TB and other TB localizations.

The Crude Incidence Rate (CIR) was calculated based on Tunisian National Institute of Statistics data and was expressed as the number per 100,000 inhabitants inh[12]. The average population was calculated as follows: (Monastir population in 2004 + Monastir population in 2014)/2. The Age-Standardized incidence Rate (ASR) was calculated using the world standard population according to the World Health Organization statement of 2013 expressed as the number per 100,000 Person year (PY) [15]. Crude death rate is an estimate of the rate at which members of a population die during a specified period.

### Statistical methods

Data were collected using Epi Info verified and analyzed using IBM SPSS Statistics version 22.0 software. Categorical variables were reported as count and percentages. Linear regression was used to calculate the slope ‘b’ of the least-squares line for estimating the trends in notified disease according to sex and age group. A p-value of <0.05 was considered statistically significant. Absolute risk /100,000 inh, Absolute Risk Reduction (ARR) /100,000inh; Relative Risk (RR), Relative Risk Reduction [16] and Number Needed to Treat (NNT) were calculated using Excel. Cox regression analysis was used to determine the hazard ratio (HR) of TB disease according to the 3 immunization protocols compared to NVC. Separate models were performed for each TB localization.

### Ethical considerations

The study was approved by the Institutional Ethics Committee of Faculty of Medicine of Monastir, Tunisia.Data were fully anonymized before analyze. Authors had not access to identifying patient information. Patients provided oral consent to have data from their medical records used in this study.

## Results

### Participants

During 18 years, 997 cases of TB were declared in Monastirwith a mean of 55.38 cases per year (95% CI:52.17; 58.60). Cases were distributed as follows, 509 EPTB(51.05%) and 486 PTB (48.95%) (p = 0.429) The most frequent sites were pulmonary localization (n = 486), lymph nodes (n = 234), pleural (n = 35) and articular-bone localizations (n = 31) (Table 1). Sex ratio was 1.22 (p = 0.001). One hundred and thirty-three cases were aged less than 20years, 399 between 20 and 39 yearsand 463 were aged more than 39 years (p < 0.0001).The median age in pulmonary localization was significantly higher (40.5 years (IQR: 28.0; 56.0)) than ganglionic localizations (30.0 years (IQR: 19.5; 56.0)) (p<0.0001). Jemmel and Monastir delegations had englobed 47.2% of cases.

**Table 1:** Frequency, age and sex ratio of reported tuberculosis cases by site of disease.

Localizations	N	%	Median age (IQR)	Sex-ratio
Pulmonary	486	48.7	40.5 (28.0;56.0)	2.86^a^
Extra-pulmonary	511	51.3		
Lymph Node	234	23.5	30.0 (19.5;47.5)	0.50^a^
Pleural	35	3.5	36.0 (27.0;62.0)	0.94
Articular-bone	31	3.1	50.0 (27.0;65.0)	0.55
Genito-Urinary	20	2	53.5 (36.0;62.5)	0.33^b^
Digestive	19	1.9	43.0 (31.0;50.0)	0.72
Ocular	7	0.7	41.0 (25.0;47.0)	1.33
Central Nervous System Meningitis	6	0.6	43.0 (15.5;66.0)	0.50
Cutaneous	4	0.4	49.5 (33.7;66.0)	0.33
Renal	1	0.1	61.0	- (δ)
Extra-pulmonary not classified **	154			

** Extra-pulmonary without reported localization (2000–2006: n = 154)

a:p<0.0001;

b: p = 0.025

### Incidence, and trends of TB

ASR per 100,000 PY was 11.53 for all sites, 5.71 for PTB and2.57 forlymph node.Males dominated in PTB (sex-ratio = 2.86 (p<0.0001)), whereas females dominated in lymph node TB (sex-ratio = 0.50 (p<0.0001)) (Table 2).

**Table 2:** Crude and age standardized incidence rates of declared tuberculosis according to age and gender (2000–2017).

	All Localizations			Pulmonary			Extra Pulmonary			Lymph node (2006–20017)		
	Cases(%)	CIR *	ASR*	Cases (%)	CIR	ASR*	Cases (%)	CIR	ASR*	Cases (%)	CIR	ASR*
**Over all**	997	10.99	11.53	486(48.7)	5.36	5.71	509(51.05)	5.62	5.82	234	2.57	2.57
**Age groups (Years)**												
0–19	133 (13.4)	3.95		34(6.99)	1.01		99(19.45)	2.94		58(24.89)	1.50	
20–39	399 (40.1)	12.95		203(41.76)	6.59		196(38.50)	6.36		100(42.91)	2.10	
40–59	286 (28.7)	15.65		150(30.86)	8.21		136(26.72)	7.44		53(22.74)	1.51	
≥60	177 (17.8)	22.62		99(20.37)	12.65		78(15.32)	9.97		22(9.44)	1.34	
**Gender**												
Men	548(54.9)	12.04	12.95	360	7.91	8.61	188(36.9)	4.13	4.33	78(33.3)	1.69	1.66
Women	447(44.8)	9.93	10.18	126	2.79	2.88	321(63.06)	7.13	7.30	156(66.7)	3.46	3.47

*: per 100,000 inhabitant/year;

**CIR**: crude incidence rate; **ASR**: age standardized incidence rate;

No patient was carrying human immunodeficiency virus in this cohort. From 2000 to 2017, a total of 11 deaths had been recorded, the fatality and crude death rate were 0.01% and 0.12/100,000 inh respectively.

We have established, an increasing trendfor all cases of TB (b = 0.58; r = 0.33; p<0.0001).A negativetrend was noted for PTB localization (b = - 0.82; r = -0.67; p<0.0001), especially in menwhile a positive trend was noticed for EPTB (b = 1.37; r = 0.78; p<0.0001) especially for lymph node localization (b = 1.31; r = 0.63; p<0.0001) (Table 3).

**Table 3:** Tuberculosis trends from 2000 to 2017 by according to age group and gender.

	All		Pulmonary		Extra pulmonary		Lymh node		Other localizations	
	Slope (SE)	r	Slope (SE)	r	Slope (SE)	r	Slope (SE)	r	Slope (SE)	r
**Over all**	0.58** **(0.05)**	0.33	-0.82**(0.04)	-0.67	1.37**(0.48)	0.78	1.31** **(0.106)**	0.63	**0.28 (0.08)**	0.26
**Age groups (Years)**										
0–19^a^	0.30**(0.03)	0.58	-0.04 (0.042)	-0.16	0.38**(0.04)	0.69	0.39**(0.09)	0.49	0.26*(0.10)	0.57
20–39 ^b^	0.075 (0.04)	0.08	-0.37** (0.042)	-0.53	0.28** (0.03)	0.50	0.85**(0.06)	0.80	-0.26**(0.06)	-0.51
40–59 ^c^	0.11 (0.06)	0.10	-0.42** (0.066)	-0.46	0.39** (0.06)	0.44	0.15 (0.10)	0.20	0.014(0.06)	-0.03
≥60 ^c^	-0.007 (0.028)	-0.02	-0.20** (0.032)	-0.53	0.22**(0.02)	0.69	0.25*(0.10)	0.49	0.36**(0.07)	0.62
**Gender**										
Men	-0.50** (0.05)	-0.35	-0.83** (0.048)	-0.67	0.25**(0.05)	0.30	0.194* (0.07)	0.27	-0.21*(0.08)	-0.32
Women	1.09**(0.02)	0.90	-0.014	-0.026	1.14**(0.03)	0.88	1.02**(0.09)	0.66	0.46**(0.05)	0.63

* p<0.05

** p<0.00001

a: corresponding to the vaccinated cohort

b: corresponding to the transient cohort that is getting vaccinated over years

c: corresponding to the not vaccinated cohort

### BCG vaccine impact

During our study period, we found364 vaccinated cases(36.5%) versus 631 not vaccinated (63.3%)(p < 0.0001).The median age was 22 years [IQR:16–27] and50 years [IQR:39–61] in VC and NVC, respectively. Sex ratio was 1.52 in NVC (p = 0.001) and 0.84 in VC (p = 0.116).

**Fig 1:**
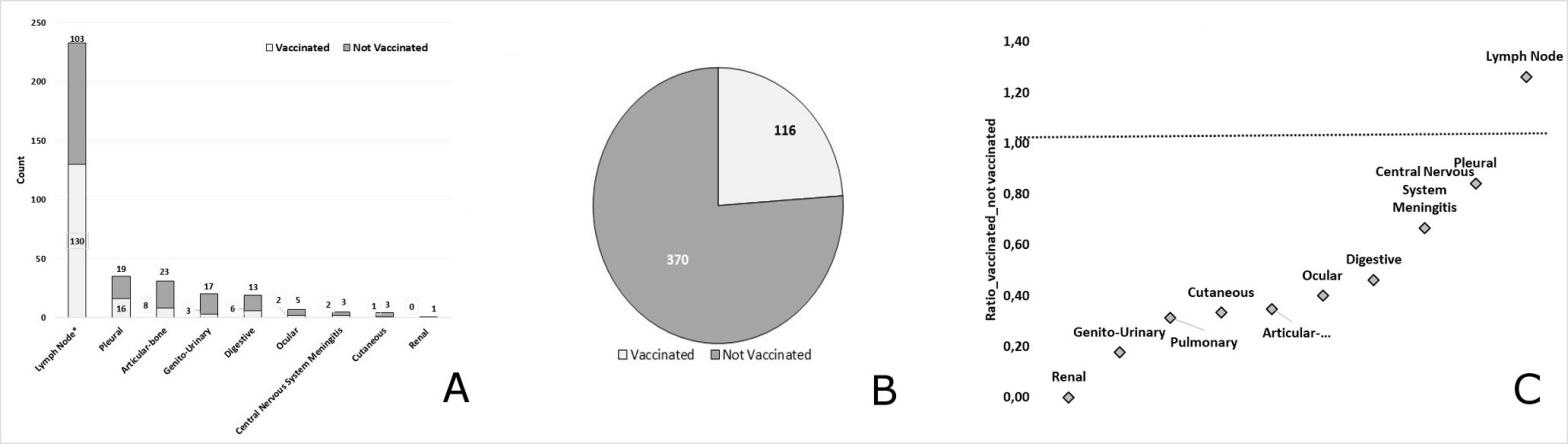
Age distribution of pulmonary tuberculosis cases according to immunization Status. In transient cohort (aged 23–37 years) a reduction of 23% of cases have been notified (VC = 67vs NVC = 88 cases).

We notified 136 PTB cases in VC (28.0%) and 350 in NVC (72.0%). Sex ratio was 4.15 in NVC (p = 0.0001) and 1.34 in VC (p = 0.086). In transient cohort (aged 23–37 years) a reduction of 23% of cases have been notified (Fig 1). CIR was 8.17/100,000 inh and 2.85/100,000 inh respectively in NVC and VC (p < 0.0001). Relative risk reduction of BCG vaccine on pulmonary localization was 65.1% (95%CI: 57.5; 71.4%). Number needed to vaccine for preventing one case of PTB per year was 18781 (15922; 22891).

In the period 2006–2017, for lymph node TB localization,138cases were not vaccinated (58.9%) versus 96 vaccinated (41.0%).Sex ratio was 0.38 in NVC (p = 0.0001) and 0.58 in VC (p = 0.02).CIR was 3.79/100,000 inh and 3.93/100,000 inh respectively in NVC and VC, (p = 0.789).

For other localizations TB, sex ratio was 0.64 in NVC (p = 0.003) and 0.73 in VC (p = 0.140). CIR was 7.39 /100,000 inh and 2.56/100,000inhrespectively among NVC and VC, (p < 0.0001). Relative risk reduction of BCG vaccination on other localizations was 65% (95%CI: 55–73%). Number needed to vaccine for preventing one case of other localizations was 20701 (16874;26774). (Table 4).

**Table 4:** Impact of BCG vaccine according to tuberculosis localization.

Years		Cases	Population	Absolute risk * (95% CI)	ARR*	Relative risk (95% CI)	RRR (95% CI)	NNT	P
Pulmonary									
2000–2017	Vaccinated	136	4770411	2.85 (2.37 ;3.33)	5.32	0.34(0.286 5 0.4)	0.651 (0.575 ;0.714)	18781(15922;22891)	< 0.0001
	Not Vaccinated	350	4281138	8.17 (7.31 ;9.03)					
Lymph node									
2006–2017	Vaccinated	138	3506529	3.93 (3.28; 4.59)	-	3.93(3.27;4.59)	-	-	0.789
	Not Vaccinated	96	2527837	3.79 (3.04;4.56)					
Other localizations									
2006–2017	Vaccinated	90	3506529	2.56(2.03;3.10)	4.831	0.34(0.27;0.44)	0.65(0.55;0.73)	20701 (16874; 26774)	< 0.0001
	Not Vaccinated	187	2527837	7.39(6.33;8.45)					

ARR: Absolute risk reduction; RRR: Relative risk reduction; CI: confidence interval, NNT: number needed to vaccine

*: per 100 000 inh

**Fig 2:**
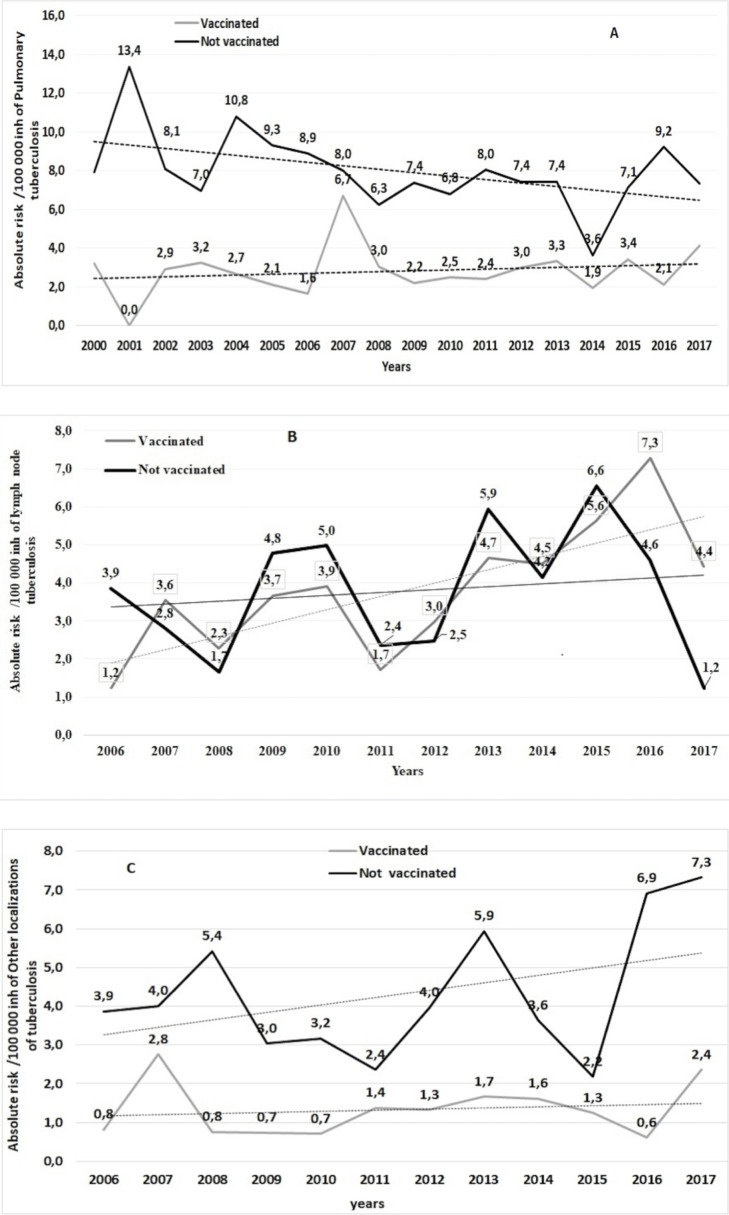
Trends of tuberculosis crude incidence per 100,000 inh according to vaccination status. A: Pulmonary TB trends: pulmonary tuberculosis (PTB) trends were stable in vaccinated cohort (b = 0.031; r = 0.12; p<0.0001) and slightly decreasing in Not vaccinated one (b = -0.14; r = -0.35; p<0.0001). B: Other localizations of TB trends: trends of other localizations TB was stable in vaccinated cohort (b = 0.07; r = 0.4; p<0.0001) and slightly increasing in not vaccinated one (b = 0,12; r = 0.29; p<0.0001). C: Lymph nodes TB trends: lymph node TB was increasing in both vaccinated (b = 0.32; r = 0.73; p<0.0001) and not vaccinated cohort (b = 0.12; r = 0.29; (p<0.0001).

PTB trends were stable in VC (b = 0.031; r = 0.12; p<0.0001) and decreasing inNVC (b = -0.14; r = -0.35; p<0.0001).Lymph nodes occurrence was increasing in both VC (b = 0.32; r = 0.73; p<0.0001) and NVC (b = 0.12; r = 0.29; (p<0.0001).Trends of other localizations TB was stable in VC (b = 0.07; r = 0.4;p<0.0001) and slightly increasing in NVC (b = 0.12; r = 0.29; p<0.0001)(Fig 2).

### The three immunization protocols

Protocol 3 had the highest effectiveness on all TB localizations with an RRR of 96.7%(95%CI: 86.6%; 99.2%),86% (95%CI:71%;91%) and 43%(95%CI:2.9%;67.3%)among PTB, other localization and lymph node TB consecutively. According to our database, protocol 1 and 2 had no significant effect on lymph node TBrisk reduction (Table 5).

**Table 5:** Impact of BCG vaccine according to protocol adopted and to tuberculosis localization.

		Cases	Population	Absolute risk * (95% CI)	ARR*	Relative risk (95% CI)	RRR (95% CI)	NNT	P
Pulmonary									
P1	Vaccinated	121	2749050	4.16 (3.42;4.90)	4.01	0.51 (0.41;0.63)	0.49 (0.37;0.59)	24911 (19158;35605)	< 0.0001
	Not Vaccinated	350	4281138	8.17 (7.31 ;9.03)					
P2	Vaccinated	13	1320622	0.93 (0.42;0.14)	7.24	0.11 (0.065;0.198)	0.88 (0.80;0.93)	13803 (11400;17489)	< 0.0001
	Not Vaccinated	350	4281138	8.17 (7.31 ;9.03)					
P3	Vaccinated	2	700738	0.26 (-;0.06)	7.9	0.033 (0.008;0.132)	0.967 (0.868;0.992)	12649(10030;17119)	< 0.0001
	Not Vaccinated	350	4281138	8.17 (7.31 ;9.03)					
Lymph node									
P1	Vaccinated	92	1834184	5.01(3.99;6.04)	-	1.32(0.992;1.758)	-	-	0.056
	Not Vaccinated	96	2527837	3.79(3.03;4.55)					
P2	Vaccinated	31	971038	3.19(2.06;4.32)		0.84(0.561;1.260)			0.4
	Not Vaccinated	96	2527837	3.79(3.03;4.55)					
P3	Vaccinated	15	701305	2.13(1.05;3.22)	1.65	0.56(0.327;0.971)	0.436(0.029;0.673)	60286 (31156;926799)	0.038
	Not Vaccinated	96	2527837	3.79(3.03;4.55)					
Other localizations									
P1	Vaccinated	70	1834184	3,81(2.92;4.71)	3.582(3.580;3.583)	0.51(0.39;0.67)	0.48(0.32;0.61)	27926(19841;47131)	< 0.0001
	Not Vaccinated	187	2527837	7.39(6.33;8.45)					
P2	Vaccinated	13	971038	1.33(0.61;2.07)	6.053(6.052;6.054)	0.180(0.103;0.318)	0.81(0.682;0.897)	16506 (12775;23314)	< 0.0001
	Not Vaccinated	187	2527837	7.39(6.33;8.45)					
P3	Vaccinated	7	701305	0.99(0.25;1.74)	6.395(6.394;6.397)	0.134(0.063;0.287)	0.86(0.71;0.93)	15627 (11835;22995)	< 0.0001
	Not Vaccinated	187	2527837	7.39(6.33;8.45)					

ARR: Absolute risk reduction; RRR: Relative risk reduction; CI: confidence interval; NNT: number needed to vaccine P: protocol; Protocol 1, (1979 to 1995):first dose at birth followed by other injections at 6, 12 and 18 years of age; Protocol 2, (1996 to 2005), vaccinations at 12 and 18 years old were removed from the program. Protocol 3:(2006 to 2017) the 6-year injection was also removed; a single dose is administered at birth.

*: /100 000 inh

**Fig 3:**
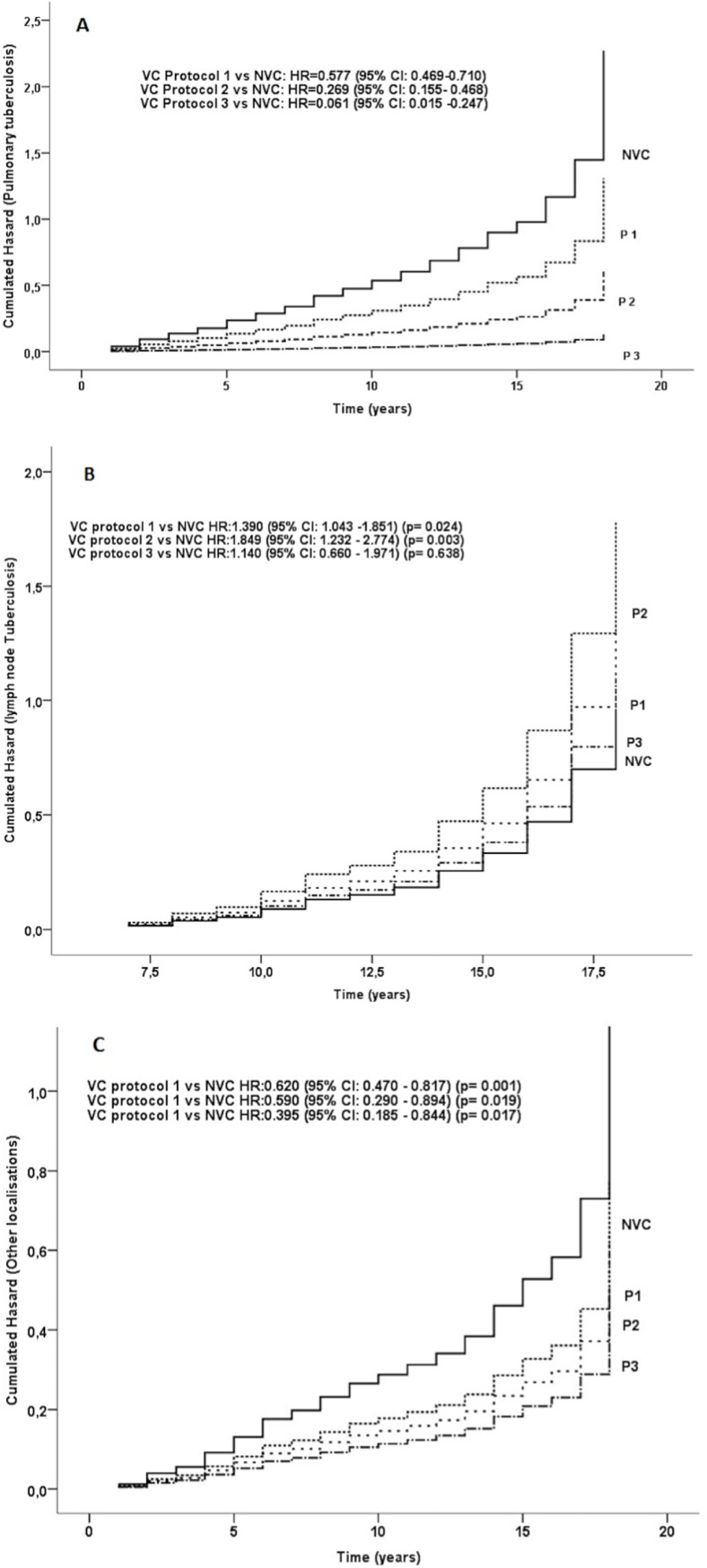
Hazard ratio of tuberculosis disease according to localizations and the three immunizations protocols. A: The three protocols were protective for developing pulmonary tuberculosis. Protocol 3 was the most effective. B: Protocols 1 and 2 were associated with an increasing risk of developing lymph node Tuberculosis. C: The three protocols were protective for developing pulmonary tuberculosis. Protocol 3 was the most effective. In protocol 1, (1979 to 1995), the first dose was administered at birth followed by other injections at 6, 12 and 18 years of age. In protocol 2, (1996 to 2005), Immunizations at 12 and 18 years old were removed from the program. Protocol 3; (2006 to 2017), the 6-year injection was also removed; a single dose is administered at birth.

Interestingly, compared to NVC, the lowest HR was observed in patients receiving protocol 3 with a HR of 0.061 (95% CI 0.015–0.247)for PTB and a HR of 0.395 (95% CI 0.185–0.844) for other localizations TB.For lymph-node TB, HR was1.390 (95% CI 1.043–1.851) for protocol 1 and 1.849 (95% CI 1.232–2.774) for protocol 2 compared to NVC (Fig 3).

## Discussion

### Key results

BCG vaccine was effective on PTB and other localizations of TB. From protocol 1 to protocol 3, it had a significant and an increasing impact on PTB incidence. A herd effect among NVC for developing PTB was established. BCG vaccinewas not effective on lymph node TB localization. Sex ratio became equally distributed among VC for all TB sites except for lymph node TB, in which it was predominantly significant in women.

### Interpretation

The ASR of TB in Monastir was 11.53/100,000 inh, less than the national level estimated at 38/100,000[8] and considered as a low incidence rate [17].TB ASR was 70 and 90 /100,000 inh in Algeria and Morocco respectively. It was also 9/100,000 inh in France and 204/100,000 inh in India[18].Our data show a PTB ASRof 5.71/100,000 PY. This rate ranged from 45 to 799 per 100 000 in the Asia-Pacific region and from 160 to 462 per 100 000 in Africa[19]. According to an Ethiopian study, lymph node TB ASR was 58/100,000 PY[20]very higher than Monastir rate. This difference can be related to socio-economic levels, justifying the social discrimination of TB. In fact, poverty is significantly linked to a higher risk of developing TB, and Monastir governorate is recognized as one of the richest cities in Tunisia.

We established that EPTB rate was equal to PTB in Monastir, in line with what was reported in Afghanistan and Qatar, with a percentage of 54.9% and 53.6% respectively [21][22]. However, studies from Turkey have noted a predominance of PTB [23]. The emergence of EPTB in recent years could be explained by the accessibility to diagnosis medical imaging techniques in our country. This has allowed the discovery of asymptomatic and latent forms.

Furthermore, the proportion of notifications classified as extra-pulmonary diseases has increased in the wake of HIV epidemic. HIV associated immunosuppression changed TB symptoms with an infection dissemination and development of extra-pulmonary manifestations [24]. PTB was the highest among males compared to females, which may be related to high tobacco consumption in this population. Smoking cause immune system altering, modifications of muco-ciliary clearance and structural lung functions changes, leading to PTB [25]. EPTB was greatest among women: The reasons for this preponderance are not visibly known, whereas, it is suggested that endocrine factors could be responsible [26].Vitamin D deficiency among Tunisian women is a modifiable risk factor [27], which may also play role in high female EPTB incidence. It contributes in boosting immune system. According to many studies, low serum vitamin D levels have been associated with a higher risk of active TB [28]. The most frequent site of EPTB was lymph nodes followed by pleural and osteo-articular TB. The site of organ involvement in EPTB varies from study to study. Some studies show that lymph node was the most frequent form of EPTB [21,22].However, in other studies, pleural were found to be involved most frequently [29,23].Reports from Hong Kong showed that genitourinary system and skin to be the commonest involved sites [30].

In both forms, TB was the lowest through the youth and the highest through elderly. It is estimated that associated diseases of elderly and comorbidities such as high blood pressure and diabetes encouraged reactivation of TB [31]. BCG vaccination policy at birth could justify the low incidence of TB among youth.

Lymph node TB was involved in almost half of EPTB cases of our distribution and the positive trend of lymph node TB from 2006 to 2017 could be explained by the high consumption of raw and unpasteurized milk, playing a key role in the transmission of MycobacteriumBovis[32]. According to the ministry of health, Mycobacteriumbovis is involved in 78.9% of all documented cases of ganglionic TB. This was attributed to a delay in implementing a national program to control infected animals.

The PTB decrease over years may be a sign of TB control program effectiveness in Tunisia and lend weight to arguments favoring the use of BCG vaccine. This could also explain the very low incidence of serious forms in Monastir. Our study showed a trend of PTB towards decline. This could be explained bythe improvement of socio-economic conditions.It has been found that, the selective vaccination reduces incidence among unvaccinated cohorts by conferring indirect protection [33], but this immunity effect was not found in our study.

According to our study, vaccination protects from PTB and other TB localizations except lymph node. Only protocol 3 showed a protective effect from lymph node TB, this could be explained by an excessive immune system response to revaccination by BCG derived from Mycobacterium bovisin protocols 1 and 2.

BCG vaccination has been used globally for protection against childhood and disseminated TB, however its efficacy at protecting against PTB in adults and agingpopulations is highly variable [34]. Our data showed a protection from pulmonary and other localizations TB of 87% and 73% respectively. Based on a meta- analysis of 14 trials and 12 case-control studies of BCG efficacy, protective effect was 50% against PTB. Protective effect from meningitis and disseminated TB were 64% and 78% respectively [35]. Vaccine effectiveness against TB varies enormously in different populations. It has ranged from 2% to 90% [35]. Indeed, BCG efficacy varied from important protection in the UK MRC trial (RR 0.22; 95%CI,0.16–0.31) [36] to absence of benefit, in a south Indian study (RR,1.05; 95%CI, 0.88–1.25) [37]. Some explanations to variations in BCG efficacy include the use of different strains and genetic reduced virulence of some M. tuberculosis strains [35].However, an analysis of trials, including 18 studies showed that the average effect of BCG vaccination was similar for each strains group. Thus, there is no evidence that the efficacy of BCG was associated with vaccination strains[36,38]. This highlights the importance of the human genetic pool, nutritional differences and the environment in generating protection [34].Cellular immunity is responsible for the protection from TB disease. Vaccination with BCG leads to the expansion of both classical antigen specific CD4+ and CD8+ T cells as well as non-classical- cells restricted T cells [39]. Genetic defects in the IFN-γ and IL-12 pathways, T cell, NK cell, monocytes and dendritic cells defects are associated with a vulnerability to mycobacterial disease [40]. Crude death rate of TB in Monastir was 0.12/100,000 inh. This rate was greater than the national level estimated at 0.04/100,000 inh but lower than international rates. Indeed, TB mortality in 2013 worldwide was 16/100,000 inh[41]. It may be explained by the higher incidence of TB in Monastir among elderly. Advanced age was found to be associated with a higher risk of TB death due to the presence of comorbidities and an aging immune system in this group [42]. Moreover, mortality could also be attributed to the increase of anti-tuberculosis drug resistance over years. In fact, chemo-resistance incidence in Tunisia was 1.14% and 31% among new and old cases respectively in 2002 [6].

Our results showed that protocol 3 was more protective than protocol 1 and 2in contrary to any additive protective effect. In fact, multiple BCG vaccination showed less significant reduction in TB disease. However, due to the recent introduction of protocol 3, our data were unable to notify TB disease cases among elderly. Thus, a longer follow up is indeed required to arrive at definitive conclusions.

Potential limitations of this study need to be considered. First, the collected data were based on passive surveillance system, therefore underreporting and lack of completeness are inevitable. Also, not declaring subgroups of extrapulmonary TB before 2006 could be responsible of an information bias.

## Conclusion

TB incidence has been considerably decreasing from 2000 to 2017underlying the efficacy of BCG vaccine in our country. Although, disease control and surveillance system should be strengthened in order to achieve the world health organizationgoal and so to end the TB epidemicby 2030.

## Supporting information

S1 Dataset Measurements for tuberculosis with source data reference. (XLS)

## List of abbreviations

BCG: Bacille-Calmette-Guérin
TB: tuberculosis
PTB: pulmonary tuberculosis
CIR: crude incidence rates
ASR: age standardized rates
RR: relative risk
VC: vaccinated cohort
NVC: not vaccinated cohort
Inh: inhabitants
PY: person years
HIV: human immunodeficiency virus
RDPH: The Regional Direction of Primary Health of Monastir
NNT: number needed to treat
ARR: absolute risk reduction
