# Correction: BCG vaccination and tuberculosis prevention: A forty years cohort study, Monastir, Tunisia

**DOI:** 10.1371/journal.pone.0223903

**Published:** 2019-10-10

**Authors:** The PLOS ONE staff

An incorrect version of Fig 1 was published in error. The publisher apologizes for the error. The authors have provided a corrected version here.

**Fig 1:**
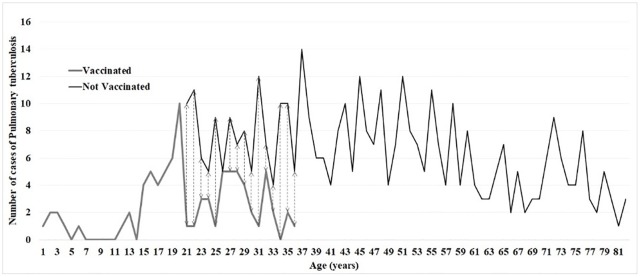
Age distribution of pulmonary tuberculosis cases according to immunization Status. In transient cohort (aged 23–37 years) a reduction of 23% of cases have been notified (VC = 67vs NVC = 88 cases).
